# Multimodal MRI Image Fusion for Early Automatic Staging of Endometrial Cancer

**DOI:** 10.3390/s25092932

**Published:** 2025-05-06

**Authors:** Ziyu Zheng, Ye Liu, Longxiang Feng, Peizhong Liu, Haisheng Song, Lin Wang, Fang Huang

**Affiliations:** 1 Informatization Construction and Management Department, Huaqiao University, Quanzhou 362021, China; zhzy@hqu.edu.cn; 2 School of Physics and Electronic Engineering, Northwest Normal University, Lanzhou 730070, China; liuyeauto@163.com (Y.L.); songhs@nwnu.edu.cn (H.S.); 3 College of Medicine, Huaqiao University, Quanzhou 362021, China; lxfengvon@gmail.com; 4 School of Engineering, Huaqiao University, Quanzhou 362021, China; pzliu@hqu.edu.cn; 5 Department of Radiology, Quanzhou First Hospital Affiliated to Fujian Medical University, Quanzhou 362000, China; 6 Radiology Department, The Second Affiliated Hospital of Fujian Medical University, Quanzhou 362000, China

**Keywords:** endometrial cancer, transformer, deep learning, MRI multi-position, early staging

## Abstract

This magnetic resonance imaging multimodal fusion study aims to automate the staging of endometrial cancer using deep learning and to compare the diagnostic performance of deep learning with that of radiologists in the staging of endometrial cancer. This study retrospectively investigated 122 patients with pathologically confirmed early EC from January 1, 2025 to December 31, 2021. Of these patients, 68 were in the International Federation of Gynecology and Obstetrics (FIGO) stage IA, and 54 were in FIGO stage IB. Based on the Swin transformer model and its proprietary SW-MSA (shift window multiple self-coherence) module, magnetic resonance imaging (MRI) images in each of the three planes (sagittal, coronal, and transverse) are cropped, enhanced, and classified, and fusion experiments in the three planes are performed simultaneously. Selecting one plane for the experiment, the accuracy of IA and IB classification was 0.988 in the sagittal, 0.96 in the coronal, and 0.94 in the transverse position, and classification accuracy after the fusion of three planes reached 1. Finally, the automatic classification method based on the Swin transformer has an accuracy of 1, a recall of 1, and a specificity of 1 for early EC classification. In this study, the multimodal fusion approach accurately classified early EC. It was comparable to what a radiologist would perform and simpler and more precise than previous methods that required segmenting followed by staging.

## 1. Introduction

**Figure 1:**
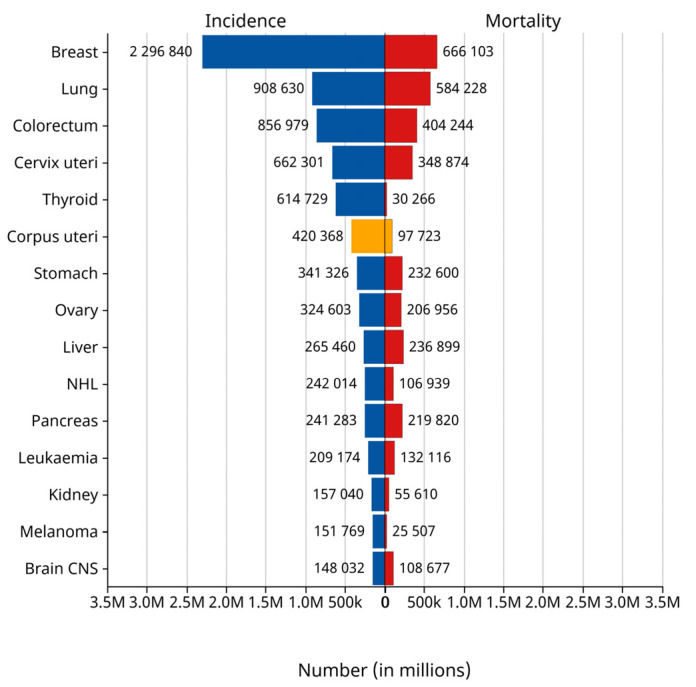
Female cancer cases worldwide by 2022. The orange represents the incidence and mortality of uterine cancer.

Endometrial cancer arises from the endometrium (the lining of the uterus or womb) [1]. It results from abnormal growth of cells that can invade or spread to other parts of the body. The first sign is most often vaginal bleeding not associated with a menstrual period [2]. Other symptoms include pain with urination, pain during sexual intercourse, or pelvic pain [3]. Endometrial cancer occurs most commonly after menopause. It is the fourth most commonly diagnosed cancer in women and the second most common malignant tumor of the female reproductive system. According to the statistics of the International Agency for Research on Cancer (IARC) of the World Health Organization in 2024, the number of new cases of endometrial cancer reached 420,000 worldwide in 2022, while 97,000 people died of the disease. Endometrial cancer ranked sixth among the cases of female tumors worldwide in that year and third among female reproductive tract tumors, as shown in the yellow bar graph in Figure 1 [4]. The EC incidence rate has increased about 1% per year since the mid-2000s among women aged 50 years and older and nearly 2% yearly since at least the mid-1990s in younger women [5]. The early stages of endometrial cancer can be categorized into stage IA and stage IB according to the Federation of Gynecology and Obstetrics (FIGO) staging [6]. For EC, the prognosis of patients in the early stage is relatively optimistic [7]. In Western countries, endometrial cancer has ranked first in the incidence of malignant tumors of the female reproductive system. Especially in the United States, it has become one of the few cancers with increasing incidence and mortality [8]. Due to the disease being accompanied by irregular vaginal bleeding, inflammation, and other clinical manifestations in the early stage [9], endometrial cancer is usually diagnosed in stage I when the tumor is confined to the uterine. Making an accurate staging prediction plays a crucial role in the treatment and prognosis of the patient [10]. The leading treatment option for endometrial cancer is abdominal hysterectomy (the total removal by surgery of the uterus), together with removal of the fallopian tubes and ovaries on both sides, called a bilateral salpingo-oophorectomy. In more advanced cases, radiation therapy, chemotherapy, or hormone therapy may also be recommended [11]. If the disease is diagnosed early, the outcome is favorable, and the overall five-year survival rate in the United States is greater than 80% [12].

MRI has long been recognized as a useful preoperative diagnostic tool and is considered superior to ultrasound and CT in assessing the depth of myometrial and cervical infiltration [13,14]. MRI can show tumor size, indicate the extent of myometrial infiltration or cervical extension, detect enlarged lymph nodes suspected of metastasis, and identify tumors that invade adjacent organs and spread to distant organs. Due to the characteristics of multi-parameter, multi-sequence, multi-directional imaging, and high tissue resolution, MRI can comprehensively display the anatomical structure and signal characteristics of the uterus and surrounding tissues [15,16]. Therefore, the importance of MRI examination lies not only in the accurate localization and qualitative diagnosis of endometrial cancer by MRI but also in clearly showing the infiltration depth, extent, and lymphatic metastasis of the tumor, and, thus, the tumor can be accurately staged [17]. MRI can guide the choice of clinical surgery and the postoperative evaluation. MRI is the best imaging method for displaying the female pelvic structure due to its numerous advantages. For endometrial cancer, MRI is necessary for staging [18]. The value of multimodal MRI is especially important in stage I, where patients have a 5-year survival rate of up to 90%. Early diagnosis and staging difficulty are limited to detecting small endothelial lesions and superficial muscle invasion. Deep learning demonstrates significant advantages over traditional machine learning techniques. It eliminates the tedious step of manually extracting image features. Instead, it relies on automatic learning mechanisms within the network to recognize features, thus greatly reducing the reliance on a deep background of specialized knowledge.

With the development of deep learning, more and more radiologists are beginning to analyze the MRIs of EC patients using deep learning methods. In the study by Jia et al [19,20], 4252 features from three sequences of T2WI, CE-T1WI, and ADC for each patient were used and tested under five machine learning models. Support vector machines performed best and were validated in both the internal test cohort and the external validation cohort. The corresponding AUCs were 0.875 (95% CI, 0.762–0.988) and 0.862 (95% CI, 0.781–0.942), respectively. However, although three sequences were used throughout the process, only the axial positions were selected, which did not allow a comprehensive analysis of the lesions. The process of manually delineating the ROIs was labor-intensive [21]. Teresa et al. used T2-weighted images (T2WI) and fused diffusion-weighted images (DWI) to assess the depth of myometrial invasion. Both results were compared with histopathological assessment. When comparing the two sets of images (T2WI and fused T2WIDWI) for the diagnosis of myometrial invasion, the fused images had a higher accuracy, and the difference was statistically significant (*p* < 0.001). The diagnostic accuracy rate for the T2WI analysis was 82.1% (70.6–88.7), whereas the fused images were diagnostically correct at 92.1% (79.5–97.2). However, they were also an orientation of the sequence chosen for the long axis of the uterine body [22].

This study proposes a Swin transformer-based framework for the early staging of endometrial cancer with multi-body position fusion. The framework firstly extracts the tumor feature information from three different body positions of the patient’s MRI sequence; then, a spatial multi-axial self-attention mechanism is constructed to capture the contextual connections while preserving the positional information, which optimizes the feature characterization and reduces the computation amount; and finally, the classification vector is obtained and fed into the fully connected layer to carry out the classification, completing the early staging of endometrial cancer. Compared with other staging methods, this paper’s proposed method considers the interconnectivity of the three body positions, and the staging results are more accurate.

## 2. Materials and Methods

### 2.1. Patients

This retrospective study included 122 patients (68 stage in IA and 54 stage in IB) diagnosed with early-stage EC by postoperative biopsy pathology at The Second Affiliated Hospital of Fujian Medical University and Quanzhou First Hospital Affiliated to Fujian Medical University from 1 January 2015 to 31 December 2021. The hospital’s medical ethics approved the study, and the requirement for informed consent was waived for all participants. The patient data collected from the hospitals were stored in DICOM format. Out of the 385 patients who underwent MRI, 122 were diagnosed as stage I and had T2WI sequences in three planes. The exclusion criteria were as follows: (1) without a final pathologic diagnostic statement; (2) absence of complete T2WI sequences in all three planes; and (3) simultaneous with other cancers.

**Figure 2:**
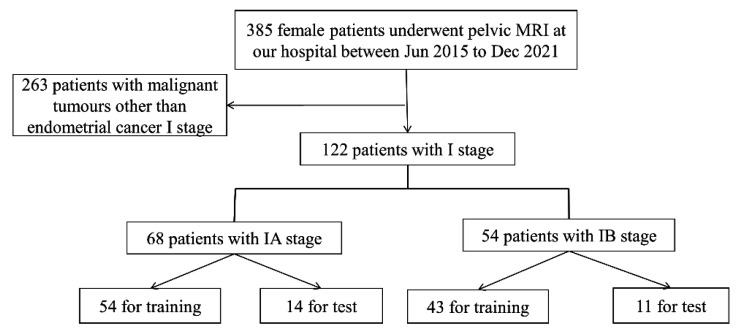
The patient selection process.

All 122 patients underwent preoperative pelvic magnetic resonance imaging with a pathological diagnosis of stage I EC (age range 28–80 years, mean age: 55.7 years, standard deviation (SD): 7.9 years). The patient selection flowchart is shown in Figure 2, and patients’ clinical and pathological data are summarized in Table 1.

The table indicates the presence of other tumors, such as clear cell carcinoma, uterine fibroids, etc.

### 2.2. MRI Examination Methods

All MRI examinations were performed using a 1.5T MRI scanner (Optima MR360, GE Healthcare, Chicago, IL, USA) and a phased-array coil. Patients were defecated with a glycerol enema before the scan and had adequate urine output (approximately half). No enemas or slowing agents were used for bowel movements to reduce bowel artifacts and motility artifacts caused by large bowel movements. Food is allowed (the food must not contain iron), and there is no need for intramuscular medication injections. It must be ensured that the patient has no contraindications to MRI and no metallic foreign bodies in the body.

### 2.3. Data Preparation and Processing

ITK-snap (version 4.0.1), a medical image reading, analysis, and manipulation software [23], was used to convert the patients’ DICOM files into image data, with 19–24 slices per sequence. The sequence used in this study was T2WI, the radiologist’s preferred reading for the patient’s MRI [24]. For 7808 MRI images (2318 sagittal, 2928 transverse, and 2562 coronal). Some images with severe artifacts were eliminated to ensure the high quality of the training data. Radiologists usually select the anterior and posterior MRI slices of the largest tumor in one or more sequences when diagnosing. Based on this feature, we can localize tumors to layers 8 to 11 in the sagittal plane, 13 to 17 in the transverse plane, and 8 to 12 in the coronal plane. This resulted in 715 sagittal slices, 645 coronal slices, and 505 transverse slices. Experimental data were randomly divided 8:2 into training and test datasets. The training dataset consisted of 97 cases (54 in stage IA, 43 in stage IB) with 1492 images (572 in sagittal position, 516 in coronal position, and 404 in transverse position). The test dataset comprised 25 cases (14 IA, 11 IB) with 373 images (143 sagittal, 129 coronal, 101 transverse). The cohort selection flowchart is shown in Figure 1.

**Figure 3:**
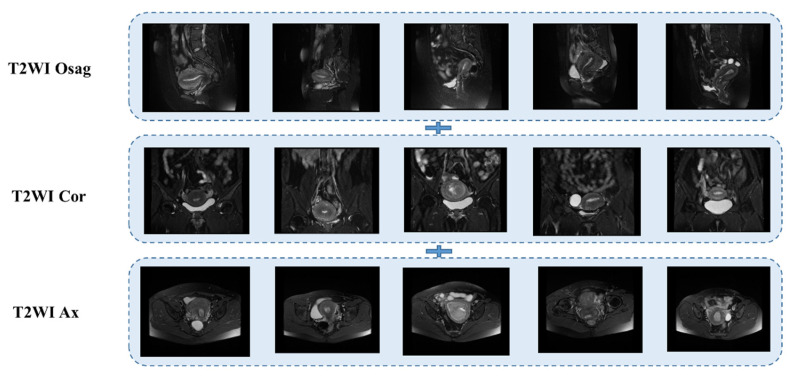
MRI T2WI sequence of endometrial cancer.

The T2WI sequence was one of the first sequences chosen by radiologists for MRI of patients with endometrial cancer [25,26]. It provides clear imaging with high resolution and allows clear differentiation of the molecular uterus, myometrium, and endometrial layers for staging. Conventional uterine T2WI can realistically show the three-layer structure of myometrium (proliferative stage—medium signal, secretory stage—high signal, postmenopausal stage 3- signal weakening and atrophy), myometrium conjugate (medial portion of myometrium, smooth muscle structure is tight, about 2~8 mm low-signal bands), and endometrial cavity (high signal). Some use a single sequence plane to extract tumor features [27,28,29]; in this case, we used three planes, the sagittal, coronal, and transverse T2WI, which are presented in Figure 3 below.

### 2.4. Deep Learning with Swin Transformer Model

**Figure 4:**
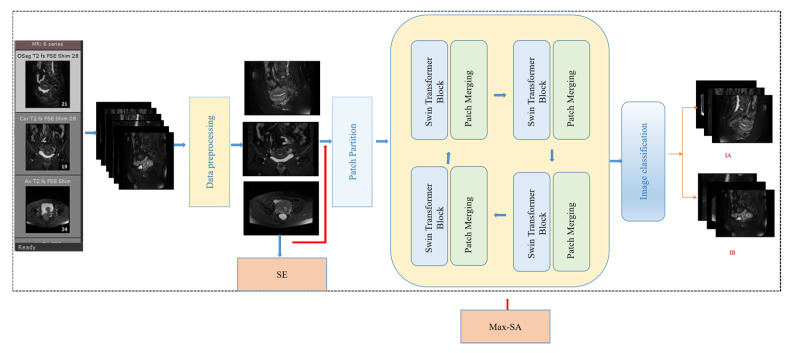
Swin transformer structure.

The transformer is a natural language processing model that exploits the attention mechanism and is crucial in developing deep learning [30]. The Swin transformer introduces a key innovation in the form of SW-MSA, which linearly solves the problem of self-attentive complexity associated with image size [31]. This enhancement simplifies the processing of high-resolution images, such as MRI images, which are often complex and high-resolution. The cancer foci usually appear as grey and low-saturated pixels in images of T2WI sequences of MRI of endometrial cancer. The enhanced SW-MSA module in the Swin transformer effectively improves self-attention and enables efficient detection of cancerous focal regions. The model structure of the Swin transformer is shown in Figure 4. The figure shows the Swin transformer block structure, where a Swin transformer block consists of a shift-window-based MSA module followed by a 2-layer MLP sandwiched between GeLU nonlinearities. A LayerNorm (LN) layer is applied before each MSA module and each MLP, and a residual bond is used after each module. LayerNorm (LN) is applied before each MSA module and MLP, with residual bonding after each module.

**Figure 5:**
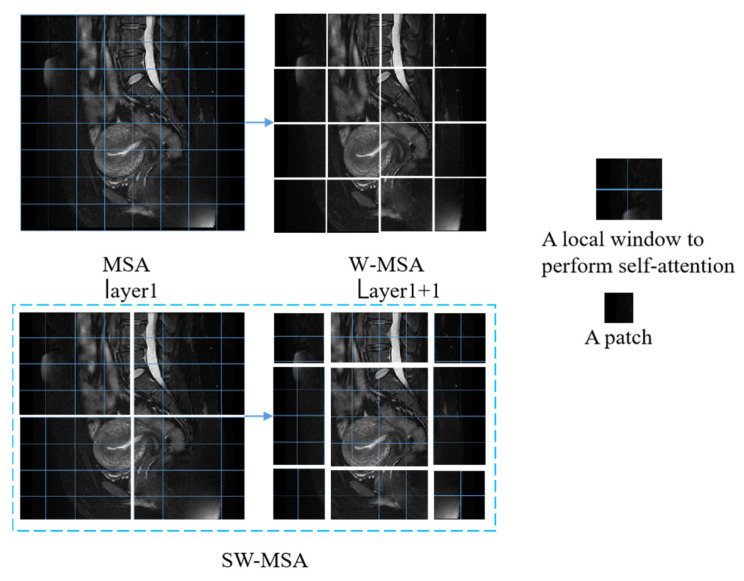
Shifted windows transformer block and sliding attention mechanism.

As shown in Figure 5, for the regular MSA module, the values of their q, k, and v are solved for each patch, and the q solved for any patch is a similar match to the other pixels k in the feature map. The disadvantage of this method is that the windows cannot interact with one another, so the detection field is reduced. Generally, W-MSA and SW-MSA are used in pairs; for example, in the bottom right corner of Figure 3, layer 1 uses W-MSA, and layer 1 + 1 uses SW-MSA, allowing information to be exchanged between windows. At the same time, there is a reduction in the amount of calculation work.

The calculation of two successive blocks of Swin transformers will be as follows:

(1) $${\hat{Z}}^{l}=W-MSA\left(LN\left(Z^{l-1}\right)\right)+Z^{l-1}$$

(2) $$Z^{l}=MLP\left(LN\left({\hat{Z}}^{l}\right)\right)+{\hat{Z}}^{l}$$

(3) $${\hat{Z}}^{l+1}=SW-MSA\left(LN\left(Z^{l}\right)\right)+Z^{l}$$

(4) $$Z^{l+1}=MLP\left(LN\left({\hat{Z}}^{l+1}\right)\right)+{\hat{Z}}^{l+1}$$

There is no position encoding module in the Swin transformer, and the position information is embedded by calculating the softmax in the attention module by adding the relative position bias B according to the following equation:

(5) $$Attention(Q,K,V)=softmax(\frac{QK^{T}}{\sqrt{d_{K}}}+B)V$$

Q, K, and V are query, key, and value matrices, and B is taken from the bias matrix.

The trained images enter the Swin transformer module and are first partitioned by the patch partition layer. The partitioned data then enter the linear embedding layer for feature mapping. The feature-mapped data enter the Swin transformer block, which, together with the linear embedding, is known as stage 1. The following 2–4 stages are downsampled to produce the slices. Finally, the output module classifies the data passing through stage 4.

The Swin transformer employs a local window-based self-attention mechanism for feature modeling. The workflow of its core component, W-MSA (window-based multi-head self-attention), can be paraphrased as follows: The input feature map (assuming a spatial resolution of H × W) is partitioned into a grid of M × M pixel units of non-overlapping windows, where the window size *M* is a fixed parameter preset by the network. The feature map is then partitioned into (H/M) × (W/M) independent computational units, with each window performing a strictly constrained local self-attention computation. This windowing processing strategy makes the computational complexity of the model present a controllable feature, which is shown in Equations (6) and (7).

(6) $$\Omega \left(MSA\right)=4hwC^{2}+2{\left(hw\right)}^{2}C$$

(7) $$\Omega \left(W-MSA\right)=4hwC^{2}+2M^{2}hwC$$

where *h* represents the feature’s height, *w* represents its width, and *C* represents its depth.

The sliding window-based transformer structure is shown in Figure 4; in the model structure diagram, shifted windows transformer block modules appear in even pairs, fusing the *W-MSA* module of the previous layer and the SW-MSA module of the latter layer. Meanwhile, Figure 5 demonstrate the image-based chunking and window-shifting strategy, where the original 8 × 8 feature map is uniformly divided into four 4 × 4 windows, each window being used for localized feature processing. Where the effect of window shifting on the feature map can be seen, each 4 × 4 window is moved in step 2 along the horizontal or vertical direction to form a new window position; this process is used to capture different local features of the image and reduce redundancy.

The Swin transformer attention mechanism faces a significant computational efficiency bottleneck when directly performing attention operations in the full spatial dimension. This bottleneck stems from the quadratic growth property of the attention operator’s space complexity. To address this challenge, this study employs a multi-axis attention decomposition mechanism, which decomposes global attention into two sparsified computational phases, local and global, through spatial axis deconstruction.

**Figure 6:**
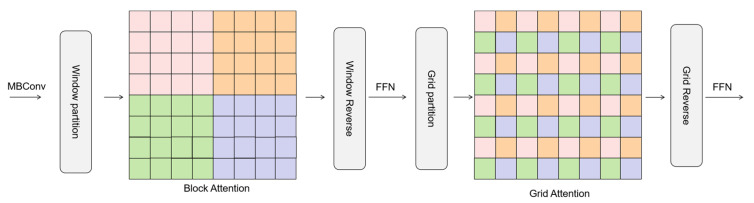
Multiaxial self-attention mechanism.

As shown in Figure 6. This operation partitions the original feature map into non-overlapping window units of (P × P) size, and this chunking strategy forms an algorithmic-level correspondence with the established mouth attention mechanism. Under this framework, the chunked attention computation inside each local window focuses on modeling the feature associations in the neighboring regions, thus efficiently enabling fine-grained spatial interactions. This multi-axis decoupled attention architecture maintains global perception capability while significantly reducing computational complexity through spatial constraints.

**Figure 7:**
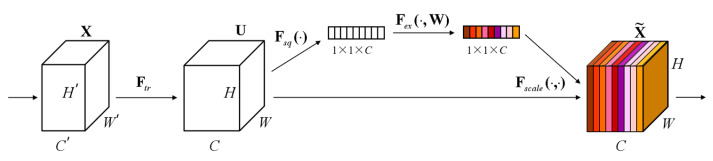
SE block structure.

At the same time, the SE (Squeeze-and-Excitation) module is added, as shown in Figure 7. The core idea of the SE Block is to learn the feature weights through the network according to the loss so that the effective feature blocks are given more weight and the ineffective or ineffective feature blocks are given less weight to train the model to achieve better results. Therefore, this study incorporates the SE module to automatically adjust the feature weights according to the loss so that the model extracts the most useful endometrial cancer staging features from the three positions.

In the squeeze stage, a global spatial aggregation strategy is used to achieve the squeeze of the null domain information and extract channel-level statistics through channel-by-channel global average pooling. Specifically, the input feature tensor X∈R^(H × W × C) is compressed in the null domain along the spatial dimension *H* × *W*. The two-dimensional feature maps of each channel are mapped into scalar form, thus generating the channel-level global description vector Z∈R^(1 × 1 × C). The process can be formally expressed as shown in (8).

(8) $$Z^{c}=F_{sq}\left(u_{c}\right)=\frac{1}{W*H}\sum_{i=1}^{W}\sum_{j=1}^{H}u_{c}\left(i,j\right)$$

where $Z^{c}$ denotes the global spatial response intensity of the cth channel; this null domain compression mechanism effectively captures each channel’s feature distribution characteristics in the receptive field’s full domain, providing a statistical a priori for the subsequent feature recalibration. The conversion of feature characterization from dense distribution in the null domain to compact encoding in the channel dimension is realized by eliminating the redundant spatial dimension information.

The excitation process is the core of SE, which is used to inscribe the weights of the C feature blocks in the tensor U. The formula is shown in (9).

(9) $$S=F_{ex}\left(z,W\right)=\sigma \left(g\left(z,W\right)\right)=\sigma \left(W_{2}\sigma \left(W_{1}z\right)\right)$$

where *σ(g(z, W))* is the Sigmoid function to achieve probabilistic compression; this weight is learned through these previously fully connected and nonlinear layers. The role of these two fully connected layers is to fuse the feature block information of each channel because the previous squeeze operates inside the feature block of a certain channel.

### 2.5. Comparison of Diagnoses with Radiologists

The 60 patient test images were independently reviewed in random order by three radiologists with 21, 16, and 4 years of experience interpreting pelvic MRI (reader 1, reader 2, and reader 3). Two are associate physicians. One is a registrar. They examined the original images to determine whether the patient was in stage IA or IB. They were blinded to the pathological and clinical results of the study. We will compare the diagnostic results of clinicians with the diagnostic results of the model and statistically evaluate the effectiveness of the depth model as a tool for early staging of endometrial cancer.

## 3. Results

We chose metrics commonly used in classification tasks, such as precision, recall, the confusion matrix, and the ROC curve [32], to express the accuracy of model detection. *TP* stands for true positive, meaning that the prediction is a positive sample and the prediction is correct. *TN* stands for true negative, meaning that a negative sample is predicted and predicted is correct. *FN* stands for false positive, meaning that a positive sample is predicted but the prediction is incorrect. Finally, *FP* stands for false negative, meaning that a negative sample is predicted but the prediction is wrong. The calculation of the accuracy, precision, recall, and specificity is shown in Equations (10)–(15).

(10) $$Precision=\frac{TP}{TP+FP}$$

(11) $$Accuracy=\frac{TP+TN}{TP+TN+FP+FN}$$

(12) $$Recall=\frac{TP}{TP+FN}$$

(13) $$Specificity=\frac{TN}{FP+TN}$$

(14) $$TPR=\frac{TP}{TP+FN}$$

(15) $$FPR=\frac{FP}{FP+TN}$$

The final results of the experiment are shown in Table 2. The selected and improved model is suitable for our experimental data, as the training accuracies for all body positions are above 0.9. The accuracy of the sagittal position is 0.99, and the accuracy of the multi-somite fusion is 1. However, the specificity of the multi-somite fusion is slightly better than that of the sagittal position. The experiment met and exceeded the expected results. The results in the table are presented after several experiments and model improvements. We chose the Swin transformer light training weight Swin-t for the experiments, and the epoch of the experiments was found to be best at epoch 50 after 20, 50, 100, and 200 experiments, and the time was also relatively short. As a rule, the larger the epochs, the better the training results, but with increasing accuracy, the loss increases and may lead to overfitting. Ultimately, we chose the higher accuracy epoch, where the loss is minimal. The trial results indicate that multi-somatic fusion is more effective than monochromatic training.

**Figure 8:**
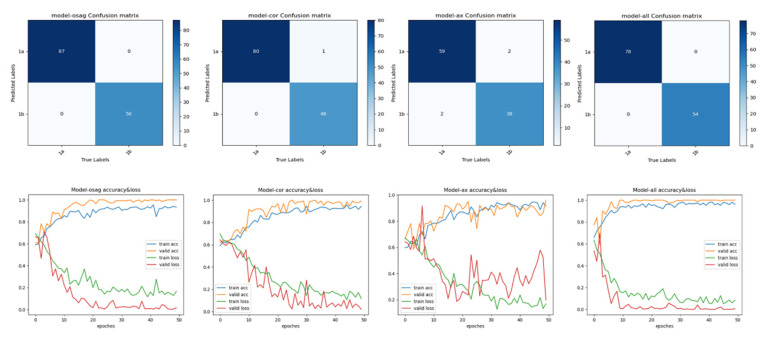
Confusion matrix and accuracy loss in different positions.

We used the loss in precision and confusion matrices to visualize the results. Figure 8 shows the confusion matrix and the accuracy loss. The top half of Figure 8 shows the results of the confusion matrix, the most commonly used metric for classifying. The positive diagonal shows the number of samples correctly classified, and the negative diagonal shows the incorrect classifications. As can be seen from the confusion matrix of the experiment, both the sagittal fusion and the multi-somatic fusion were correctly classified. There were misclassifications in both the coronal and transverse positions. The accuracy loss in the bottom half of Figure 8 shows, from left to right, the results of the sagittal training, the coronal training, and the transverse training and the results of the multi-site fusion. From the graph of the loss in accuracy, it can be seen that they all start from 0.6, and the accuracy shows an increasing trend, and the loss shows a decreasing trend. The accuracy of the sagittal position reaches 0.9 at epoch 20, the coronal position and the transverse position reach 0.9 at epoch 30, and we find that the accuracy of the multi-site fusion training reaches 0.9 at epoch 10. This shows that the multi-site fusion training converges faster. Meanwhile, the zigzag degree of the curve indicates that the training process of multi-site fusion is softer, and the effect of the transverse position is the worst.

Comparisons with some classification models, such as ViT-B/16, ResNet50, and SVM + HOT, are made in this subsection; in the four classification deep learning models explored in this section, we consistently use a uniform set of training strategies to perform the training and testing staging tasks. Specifically, we set each batch to contain eight images (the batch size is 8) and performed 300 rounds of iterative training (the number of epochs is 300) to ensure that the loss function can converge effectively. We chose Adam as the optimization algorithm for all models and set the learning rate to 0.0001. The results are shown in Table 3, where the performance metrics for each model show that the improved model Swin transformer in this chapter achieves the best performance for all metrics in the endometrial cancer multi-site fusion task. Figure 8 shows the ROC plots for the sagittal, coronal, and transverse positions, respectively, from which it can be seen that the improved Swin transformer model outperforms several other deep learning models, with multi-site fusion outperforming the other three positions in staging.

This paper designs and implements a systematic ablation experiment to investigate the contribution of each module to model performance. The experiments focused on the effects of three core modules, Max-SA multi-axis self-attention, the dynamic weight fusion SE (Squeeze-and-Excitation) module, and the hierarchical pruning strategy, on model performance. The experimental results are shown in Table 4, where the combination of modules significantly affects the model accuracy.

Performance analysis of the base model: Model (1), the base model without introducing any optimization module, has an accuracy of 0.876. This result reflects the performance benchmark of the base model without any optimization and provides an important reference for evaluating the subsequent modules.

Single-module performance impact: When the Max-SA multi-axis self-attention mechanism is introduced, the accuracy of Model (2) is significantly improved to 0.935, 6.7 percentage points higher than the base model. This result indicates that the Max-SA multi-axis self-attention mechanism can effectively capture the global contextual information and local detailed features in the image, thus significantly improving the feature extraction capability of the model.

Performance analysis of dual-module combination: In Model (3), the Max-SA multi-axis self-attention mechanism and the SE module are introduced simultaneously, and the model accuracy is further improved to 0.947. This result indicates that the SE module can adaptively adjust the weight distributions of the different feature layers, which creates a good synergy effect with the Max-SA multi-axis self-attention mechanism. Model (4) examines the Max-SA multi-axis self-attention mechanism, which can capture the image’s global context information and local details, thus significantly improving the feature extraction capability; on the other hand, examining the effect of combining the Max-SA multiaxial self-attention mechanism with medical prior knowledge (MPK), it achieves an accuracy of 0.978. This significant enhancement suggests that medical a priori knowledge provides domain-specific constraints and guidance for the model, that the a priori information of anatomical structures helps correct staging errors, and that clinical knowledge enhances the interpretability and reliability of the model. Notably, Model (5), which only used the SE module and medical priori knowledge, resulted in an accuracy of 0.953. Although this result is superior to that of a single module, there is still a gap compared to the combination that includes the Max-SA multiaxial self-attention mechanism, highlighting the attention mechanism’s critical role in feature extraction.

Performance analysis of the full-module combination: Model (6) simultaneously incorporates the Max-SA multiaxial self-attention mechanism, the SE module, and medical prior knowledge and achieves a perfect performance of accuracy = 1.0. This result fully demonstrates that the Max-SA multi-axis self-attention mechanism provides a multi-level feature representation, the SE module realizes adaptive integration of features, and the medical a priori knowledge enhances the interpretability and reliability of the model.

**Figure 9:**
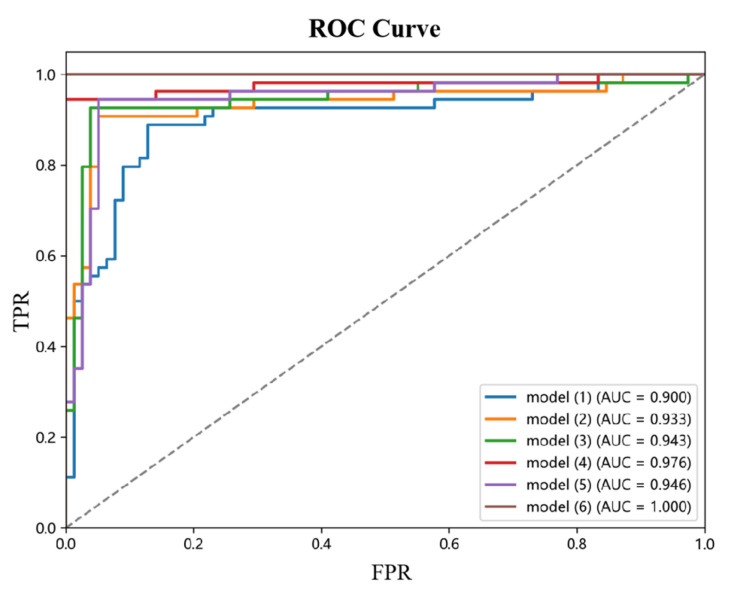
ROC for different ablation models.

Combining the three modules fully exploits their respective advantages and generates significant synergistic effects, ultimately improving model performance overall. This finding provides important guidance for subsequent model optimization. Figure 9 shows the ROC curves of different ablation models.

## 4. Discussion

**Figure 10:**
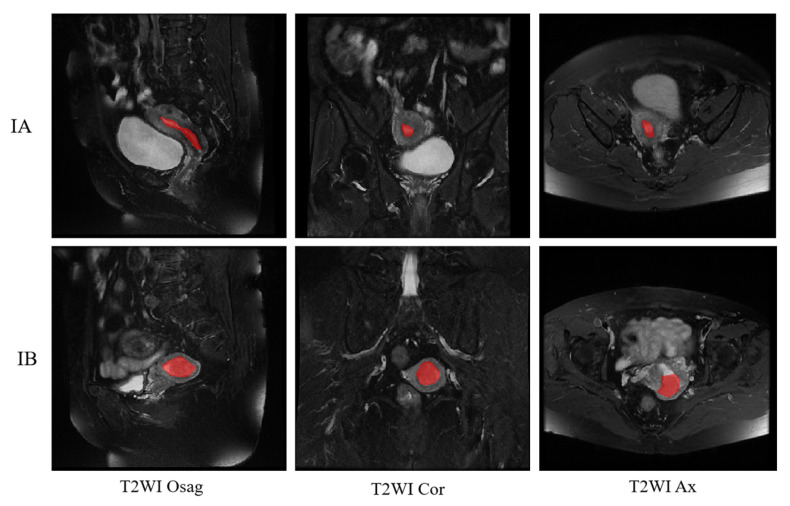
Classification results of T2WI for stage IA and IB. The red represents the cancerous area.

Figure 10 shows the final staging results. The upper side shows the segmentation image of the three positions of T2WI of a patient with stage IA, and the lower side shows the segmentation of the three positions of a patient with stage IB, where the red is the region of cancer foci. The segmentation image shows that the model correctly recognizes the cancerous areas of the three body positions.

There was also a comparison of the results of three radiologists of different grades and experience with the results of the model. The statistical results are shown in Table 5. A total of 30 patients each were externally validated for IA and IB. The model finally classified two patients from IA to IB and correctly classified all 30 patients from IB. The two radiologists at the associate director level would have misclassified both IA to IB and IB to IA, and the registrar would have misclassified one or two more patients than the director.

Deep learning, which is particularly suited to image and speech recognition, is a new research direction in machine learning that can automatically learn the intrinsic laws and symbolic levels of sample data. It has been widely used in medical-assisted diagnosis in recent years due to its excellent image-processing capabilities [33,34,35]. As one of the three most common gynecological tumors in women, endometrial cancer is becoming increasingly common at younger and younger ages. Early staging of this disease is crucial. Early staging and diagnosis are very helpful for young patients who want to preserve their fertility [36]. At the same time, the different stages of endometrial cancer lead to other surgical methods and prognoses. Therefore, it is important to use deep learning to stage endometrial cancer at an early stage.

On one hand, our research shows that multi-position fusion can facilitate the model to extract more features and perform better staging than a single position. On the other hand, our model showed slightly better diagnostic performance than the two associate physicians compared to three radiologists. The statistical results showed that the model would misclassify two patients with stage IA as stage IB and that stage IB was correct. However, radiologists have misdiagnosed both. The size of the uterus and a person’s age and whether they are menopausal or not have an effect on the thickness of the endometrium, and the cancer foci in stage I patients are inside the uterus and usually around the endometrium. Stage IA and IB are classified according to whether the infiltration of the myometrium reaches 50%, but the machine is not as accurate for patients at the critical point. The radiologists are better in this respect; they can do an excellent job of classification based on years of experience. However, the radiologists will show that IA is classified as IB and some IB is classified as stage II. Because I told the doctors at the time of the experiment that they could only grade II, they did not grade some IB as IA, but the doctors still thought some IB patients were already stage IB based on their experience and understanding of what that means. Cervical invasion and should be grade II. Although deep learning for medical imaging is not mixed with human factors and can be as objective as possible, its shortcomings are undeniable. It does not have a global view and experience in different cases compared to the doctor. Therefore, rather than replacing the doctor who ultimately makes the diagnosis, our proposed model is more of an aid. Deep learning can significantly reduce a physician’s time to read a chart, improving efficiency.

Our study differs from previous studies in that, firstly, there is no need for extensive experience and manual labeling of tumors for segmentation. Instead, it directly and automatically extracts image features relevant to lesion detection. This eliminates the need for extensive time spent on manual labeling. Secondly, most previous studies have been performed using a single-body position. We used a three-body fusion of MRI scans, which also means that the extraction of the lesion will be more comprehensive and accurate, and it also provides some basis for the accuracy of staging. Thirdly, we compared and validated with radiologists of different experiences and finally reached a level comparable to that of an associate physician.

At the same time, there are still some areas for improvement in our research work: Firstly, our sample of data is relatively small; we aim to build up an extensive database of endometrial cancer for future studies, and we are also actively collecting data from other hospitals. Secondly, although the T2WI sequences we have chosen are said to be the first sequences doctors choose for diagnosis, doctors usually choose several sequences that are read together to ensure that the most appropriate staging diagnosis is made. Our follow-up study will also add DWI and ADC sequences for preliminary data collection.

## 5. Conclusions

In this study, the Swin transformer used for early endometrial staging was modified, and a fusion experiment was performed using three T2WI positions. The results were superior to those of independent experiments using a single position, with the modified model accurately identifying cancer features in all three positions and staging at a level comparable to that of a resident with approximately 21 years of experience. This study significantly reduces the radiologist’s reading workload and improves the radiologist’s productivity. The model will improve the accuracy of endometrial cancer staging. This will help determine the best surgical approach for patients. With the continuous development of science and technology, deep learning will likely be applied to more high-end medical diagnostic tools.

## Figures and Tables

**Table 1:** Clinical and pathological data summaries. Patients’ information (*n* = 122).

Parameter	Stage IA	Stage IB	*p* Value
Subpopulation	68	54	
Age (year)	51.5 ± 9.7	60.2 ± 8.3	
**Endometrial type ^#^**			0.721
Grade 1	41	20	
Grade 2	24	26	
Grade 3	3	8	
Myometrial invasion			
<50%	65	4	0.873
≥50%	3	50	
Mixed carcinoma			0.875
No	65	51	
Yes	3	3	

^#^ Histological grading of endometrioid carcinoma. According to the solid range of tumors, the classification criteria are as follows: Grade 1, solid growth area ≥5%; Grade 2, solid growth area accounts for 6% 50%; Grade 3, solid growth area > 50%.

**Table 2:** Different position’ experiment results (epochs = 50).

		Accuracy	Precision	Recall	Specificity
Sagittal T2WI	IA	0.99	0.988	1.0	0.98
	IB		1.0	0.98	1.0
Coronal T2WI	IA	0.96	0.967	0.95	0.967
	IB		0.95	0.967	0.95
Axial T2WI	IA	0.94	0.937	0.967	0.9
	IB		0.947	0.9	0.967
Fusion Position	IA	1.0	1.0	1.0	1.0
	IB		1.0	1.0	1.0

**Table 3:** Different models’ experiment results (epochs = 300).

Model	Sagittal T2WI	Coronal T2WI	Axial T2WI	Fusion Position
SwinT	93.5	89.1	92.6	94.5
ViT-B/16	86.2	83.2	84.3	86.3
ResNet50	90.4	85.4	88.4	90.1
SVM + HOG	77.5	73.1	76.2	82.6

**Table 4:** Ablation experiment.

Model	Max-SA	SE	MPK	Accuracy
Model (1)	——	——	——	0.876
Model (2)	√			0.935
Model (3)	√	√		0.947
Model (4)	√		√	0.978
Model (5)		√	√	0.953
Model (6)	√	√	√	1.0

**Table 5:** Test results of the model and readers.

	Model	Reader 1	Reader 2	Reader 3
IA	28	28	27	26
IB	30	28	29	27
IA→IB	2	2	3	4
IB→IA	0	2	1	3

## Data Availability

The data presented in this study are available on request from the corresponding author.
